# The m6A methyltransferase METTL14 inhibits the proliferation, migration, and invasion of gastric cancer by regulating the PI3K/AKT/mTOR signaling pathway

**DOI:** 10.1002/jcla.23655

**Published:** 2020-12-12

**Authors:** Xin Liu, Mingyang Xiao, Liang Zhang, Liuli Li, Guolian Zhu, Erdong Shen, Mingyue Lv, Xiaobo Lu, Zhe Sun

**Affiliations:** ^1^ Key Laboratory of Precision Diagnosis and Treatment of Gastrointestinal Tumors Department of Surgical Oncology and General Surgery Ministry of Education The First Affiliated Hospital of China Medical University Shenyang China; ^2^ Department of Toxicology School of Public Health China Medical University Shenyang China; ^3^ Department of Thoracic Surgery Liaoning Cancer Hospital & Institute Shenyang China; ^4^ Department of Oncology Shenyang Fifth People Hospital Shenyang China; ^5^ Department of Oncology Yueyang First People Hospital Yueyang China

**Keywords:** gastric cancer, methyltransferase‐like 14, prognosis, RNA m6A modification, tumor suppressor

## Abstract

**Background:**

*N*6‐methyladenosine (m6A) modification may participate in the regulation of occurrence and development of tumors. However, the m6A level and the potential regulatory mechanism of m6A in gastric cancer (GC) remain uncertain.

**Methods:**

RNA m6A quantification assay was conducted to detect the m6A level in GC tissues and cell lines. Methyltransferase‐like 14 (METTL14) expression in GC tissues was explored by bioinformatics and immunohistochemistry. Then, the function of METTL14 in GC cells was examined by CCK‐8, colony formation assay, wound healing assay, and Transwell assay. Besides, Western blotting was conducted to probe the PI3K/AKT/mTOR pathway and the epithelial‐mesenchymal transformation (EMT) pathway‐related gene expression.

**Results:**

The m6A modification level was decreased in GC and METTL14 was a key regulator resulting in m6A disorder in GC. METTL14 was downregulated in GC by analyzing both clinical samples and bioinformatics. METTL14 overexpression suppressed GC cell proliferation and aggression by deactivating the PI3K/AKT/mTOR pathway and the EMT pathway, respectively.

**Conclusions:**

Our findings indicate that METTL14 partakes in the biological process of GC as a tumor suppressor and may be an emerging biomarker in GC.

## Graphical abstract

METTL14 depresses GC cell progression and aggression as a tumor suppressor.

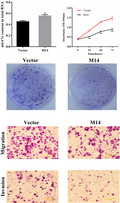

## 1 INTRODUCTION

Gastric cancer (GC), which remains a major contributor to the global burden of disease,^1^ is often diagnosed in the progressive stage. Recently, the implementation of standardized surgery for GC has ensured radical surgical treatment and has laid the foundation for improving the overall efficacy of GC treatment.^2^ At the same time, chemotherapeutic drugs, targeted drugs, and immunotherapeutic drugs have played a crucial role in GC therapy at different stages.^3^, ^4^, ^5^ However, the 5‐year overall survival (OS) rate remains low. Therefore, exploration of the key molecules and their related mechanisms may contribute to the early detection of GC and improve the early diagnosis rate; it may also aid the development of targeted drugs to improve the treatment for GC.

Post‐transcriptional regulation events have attracted increasing attention. *N*6‐methyladenine (m6A) is a modification in which a methyl group is transferred to the sixth nitrogen atom on adenine by methyltransferase complex to replace the original hydrogen atom,^6^ and it can be reversed by demethylases. Researchers have found that m6A, often found on mRNAs and long non‐coding RNAs, is widespread among most eukaryotes, such as mammals, plants, fruit flies, yeast, and some viruses, accounting for 0.1–0.4% of the total adenine population (m6A/A, approximately 3–5 m6A sites in each transcript).

Studies have confirmed that m6A can regulate alternative splicing of transcription, nuclear RNA transportation, protein translation, self‐renewal of embryonic stem cells, and circadian rhythm.^7^, ^8^, ^9^, ^10^, ^11^ In recent years, researchers found that RNA m6A modification might contribute to the occurrence and progression of tumors.^12^ For example, METTL14 deficiency reduced m6A modification level in hepatocellular carcinoma (HCC) and participated in the metastasis process of HCC.^13^ However, the m6A modification level and its potential regulatory mechanism in GC remain uncertain.

In the study, we first observed that m6A modification level was reduced in GC and METTL14, a key methyltransferase of m6A, was the regulatory factor that caused m6A modification disorder in GC. Then, in GC patients, we assessed the level and prognosis of METTL14. Furthermore, the impact of METTL14 on the biological process of GC was studied, and the potential mechanism of METTL14 in the development of GC was explored. Our study suggested that METTL14 might be an emerging biological target for the clinical diagnosis and therapy of GC.

## 2 MATERIALS AND METHODS

### 2.1 Tissue samples collection

Two hundred and forty‐eight GC tissue samples and matched adjacent tissue samples were collected from GC patients who underwent radical gastrectomy at the Department of Surgical Oncology and General Surgery of the First Affiliated Hospital of China Medical University from January 2007 to December 2008. The patients had not received preoperative chemotherapy or radiotherapy. This study was conducted according to the Declaration of Helsinki and was authorized by the Ethics Committee of the First Affiliated Hospital of China Medical University. Informed consent was obtained from all patients. Tissue samples were collected during surgery and stored in liquid nitrogen. All specimens were diagnosed by histopathology.

### 2.2 Immunohistochemical staining

Gastric cancer tissues and paired adjacent tissues were sectioned into paraffin slices after fixation, dehydration, and embedding. Immunohistochemical staining was performed as described previously.^14^ The expression levels of METTL14 in tissues were examined by two independent pathologists. The score of 0, 1, 2, and 3 represented negative, weak, moderated, and strong staining based on the staining intensity, respectively. The score of 0, 1, 2, 3, and 4 represented 0%, 1% to 25%, 26% to 50%, 51% to 75%, and 76% to 100% positive cells based on the proportion of cell staining, respectively. By multiplying the above two scores, the final score was calculated. METTL14 expression was evaluated as follows: 0–6, low expression; 7–12, high expression.

### 2.3 Cell culture

All the three GC cell lines and the normal gastric epithelial cell line (GES‐1) were ordered from the Institute of Biochemistry and Cell Biology of the Chinese Academy of Sciences (Shanghai, China). GES‐1, SGC‐7901, and MGC‐803 cells were maintained in DMEM (HyClone; Thermo Fisher Scientific, Inc) with 10% fetal bovine serum (FBS; Gibco; Thermo Fisher Scientific, Inc). AGS cells were cultured in F‐12 (HyClone) with 10% FBS. The cells were grown in a 5% carbon dioxide/ 37°C humidified incubator.

### 2.4 RNA isolation and quantitative RT‐PCR analysis

The RNAeasy™ Animal RNA Isolation Kit with Spin Column (Beyotime) was utilized to extract total RNA following the manufacturer's protocol. TB Green^®^ Premix Ex Taq™ II (Takara) and a LightCycler480 System (Roche Diagnostics) were used for qRT‐PCR. The Ct value of the gene was corrected after subtracting the Ct value of GAPDH. To calculate relative METTL14 expression, the 2^−ΔΔCt^ method was used.^15^ The primer sequences for METTL14 and GAPDH were showed in Table 1.

**Table 1:** The primer sequences included in the study

Name	Primer sequences (5′–3′)
METTL14	
Forward	GAACACAGAGCTTAAATCCCCA
Reverse	TGTCAGCTAAACCTACATCCCTG
GAPDH	
Forward	ACAACTTTGGTATCGTGGAAGG
Reverse	GCCATCACGCCACAGTTTC

### 2.5 RNA m6A quantification

The m6A RNA methylation quantification kit (ab185912; Abcam) was utilized to measure the m6A content in the total RNAs. Assay wells were coated with 200 ng RNAs. RNA m6A quantification assay was performed following the manufacturer's protocol. By measuring the absorbance at 450 nm wavelength, the m6A content was quantified.

### 2.6 Cell transfection

Lentivirus transfection was used to construct METTL14 knockdown and overexpression cell lines. The pLKD‐CMV‐mcherry‐2A‐Puro‐U6 vector was used to construct a recombinant METTL14 knockdown lentivirus, and the pRLenti‐EF1a‐EGFP‐P2A‐Puro‐CMV‐MCS‐3Flag vector was used to construct a recombinant METTL14 overexpression lentivirus. Recombinant METTLl4 knockdown lentivirus, METTL14 overexpression lentivirus, and the corresponding control lentivirus were purchased from Obio Technology (Obio Technology Corp., Ltd.). The lentivirus was transfected into GC cells following the manufacturer's protocol. The efficiency of METTL14 overexpression and interference in the stably transfected cell lines was verified by qRT‐PCR and Western blotting.

### 2.7 Cell proliferation and colony formation

The experiment was conducted according to the protocol of the Cell Counting Kit‐8 Reagent Kit (Dojindo). After the cells were maintained in a 5% carbon dioxide/37°C humidified incubator for 24 h, 48 h, and 72 h, cell proliferation was examined. A microplate reader was utilized to measure the absorbance at 450 nm wavelength. For the colony formation assay, 1,000 cells per well were seeded in 6‐well dishes for 14 days, after which they were fixed, dyed, and counted.

### 2.8 Wound healing migration assay

Cells were plated and grown in 6‐well dishes in complete medium. When the cells reached 70% to 90% confluence, a wound was scratched along the length of the culture dish with a pipette tip (200 μl). The cells were photographed by microscopy, and the size of the wound (s0) was measured. After the medium was changed to serum‐free medium, the cells were grown in the incubator for 48 h. After 48 h, the size of the wound (s48) was measured and compared with s0.

### 2.9 Transwell assay

For this assay, 24‐well Transwell chambers (3422, Corning) were utilized. For the cell migration assay, the chambers were not coated, while the chambers were coated with Matrigel (BD Biosciences) for the cell invasion assay. First, 20 000 cells in 100 μL medium without serum were added to the upper chamber, while medium containing serum but not cells was added to the lower chamber. The cells were incubated at 37°C for 24 h. Cells that did not pass through the membrane were gently removed with cotton balls, and the cells that passed through the membrane were fixed with 4% paraformaldehyde and stained with crystal violet for 15 min. Stained cells were counted in five randomly‐chosen fields under a microscope, and the mean value was calculated.

### 2.10 Western blotting

Protein exaction and Western blotting were conducted as described previously.^16^ An antibody against METTL14 (HPA038002) was purchased from Sigma‐Aldrich. Antibodies against E‐cadherin (#3195), N‐cadherin (#13116), MMP‐9 (#13667), Vimentin (#5741), *p*‐mTOR (#2971), mTOR (#2972), *p*‐PI3K (#4228), and PI3K (#4249) were ordered from Cell Signaling Technology. *P*‐AKT (AF3262) and AKT (AF6261) antibodies were purchased from Affinity Biosciences. An anti‐GAPDH antibody was used as a loading control for the experiment.

### 2.11 Bioinformatics analysis

Oncomine database (https://www.oncomine.org/), which is a publicly online tumor microarray database to facilitate discovery from genome‐wide expression analyses, was adopted to compare METTL14 expression between cancer and normal samples in GC. The searching filters were as follows: Gene name, METTL14; Analysis type, Gastric Cancer vs. Normal Analysis. With threshold by *p*‐value ≤ 1E‐4, fold change ≥1 and top 10% gene rank, two datasets: “TCGA Gastric” (http://gdac.broadinstitute.org/runs/stddata__2013_05_23/data/) and “Deng Gastric” (http://www.ncbi.nlm.nih.gov/geo/query/acc.cgi?acc=GSE31168) were included (Table S1). All datasets were log‐transformed, and standard deviations were normalized to one per array.^17^ A Student's *t* test was implemented for the comparison of tumor samples and normal datasets.

The METTL14 expression data retrieved from The Cancer Genome Atlas (TCGA) used in this study was downloaded from the UCSC Xena browser (https://xenabrowser.net). The download file of the mRNA expression matrix was IlluminaHiSeq UNC (Dataset ID: TCGA. STAD. sampleMap/HiSeqV2; Version:2017‐10‐13). Detailed TCGA sample accession numbers were provided in the Table S2. The dataset shows the gene‐level transcription estimates, as in log_2_(*x* + 1) transformed RSEM normalized count. The expression matrix includes 415 cases of primary gastric adenocarcinoma samples and 35 cases of the normal gastric tissue samples, of which 32 cases were paired samples. The expression information of METTL14 was extracted for analysis.

### 2.12 Statistical analysis

With the use of SPSS 20.0 (Chicago, IL, USA), all statistical analyses were performed. To compare data between the two groups, a two‐tailed Student's *t* test was implemented. Kaplan–Meier method and log‐rank test were used for survival analysis. To identify significant independent prognostic factors, Cox's proportional hazard regression model was utilized. All data were expressed as Mean ± SD. The results were considered statistically significant only if *p* < 0.05.

## 3 RESULTS

### 3.1 METTL14 is downregulated in GC

**FIGURE 1:**
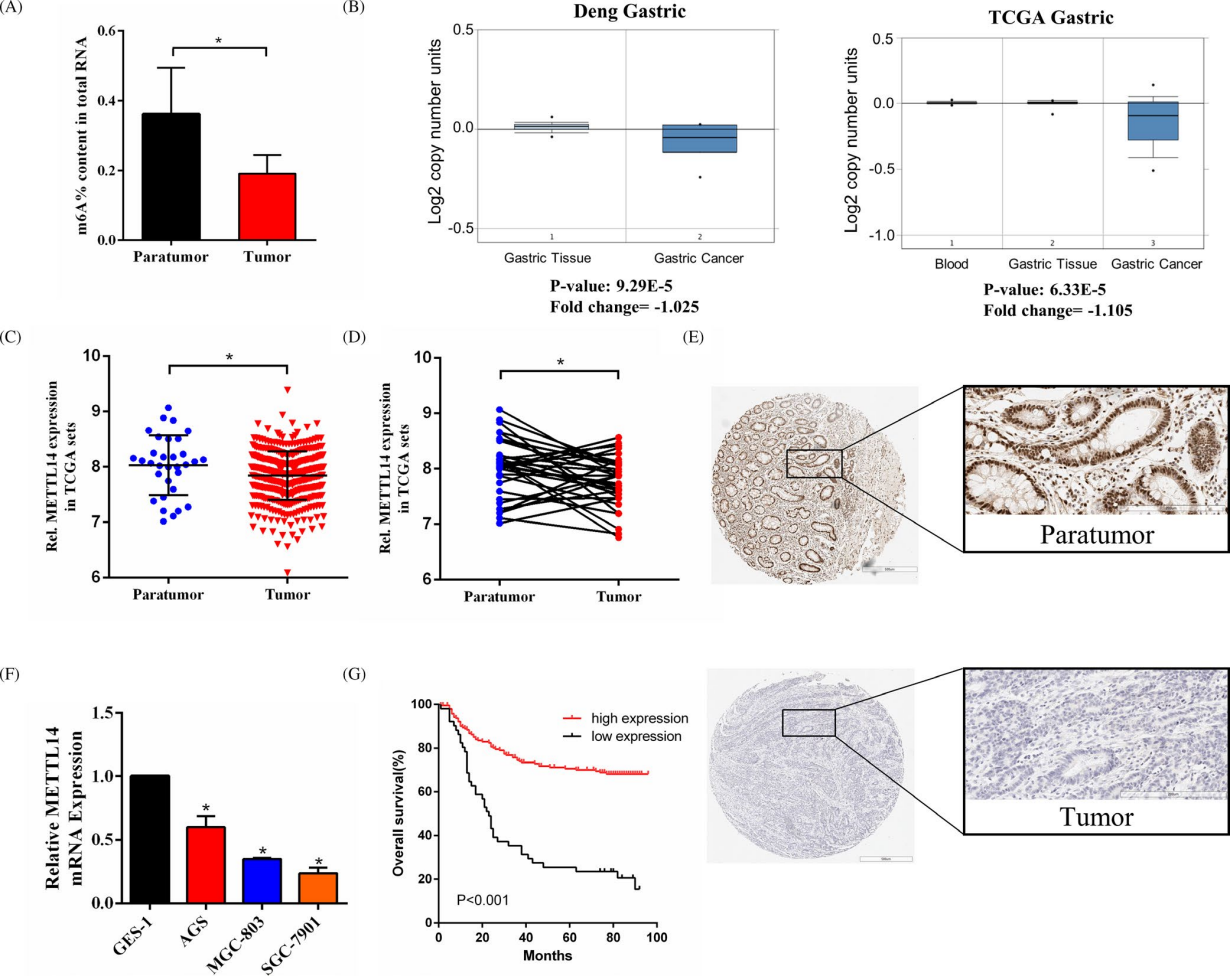
Expression of METTL14 in GC. A, The m6A contents of total RNAs in GC tissues paired with paratumor tissues. B, Bioinformatics analyses from two studies in the Oncomine database. C, In the TCGA profile, METTL14 mRNA expression in 415 GC tissues compared with that in 35 paratumor tissues. D, In the TCGA profile, METTL14 expression in 32 paired GC tissues. E, Representative immunohistochemical staining of METTLl4 in matched GC tissue and paratumor tissue. F, Relative METTL14 mRNA expression in three GC cell lines and GES‐1 determined by qRT‐PCR. G, Kaplan–Meier survival analysis. *p < 0.05

To investigate the potential role of m6A in GC, we first detected the m6A content in the total RNAs of paired GC tissues. m6A levels were decreased in GC tissues compared with that in matched adjacent tissues (Figure 1A). Methyltransferases and demethylases reversibly catalyzed RNA m6A modification. We assumed that the dysregulation of the pivotal m6A‐related factors resulted in the decreasing m6A modification in GC. To investigate our hypothesis, the Oncomine database (https://www.oncomine.org/resource/login.html) was used and the result showed that METTL14 was always downregulated in GC in two studies (Figure 1B). Using RNAseq data from the TCGA database, we then examined the METTL14 expression at the mRNA level in GC. The result showed that METTL14 was downregulated in GC tissues compared with that in peritumoral tissues (Figure 1C). Moreover, the expression level of METTL14 in 32 paired GC tissues was reviewed to further affirm the result (Figure 1D). The immunohistochemical staining was performed on paired GC tissues and indicated that GC tissues exhibited remarkably lower METTL14 expression than matched adjacent tissues (Figure 1E). We then divided the 248 GC patients into a group with low METTL14 expression and a group with high METTL14 expression based on the immunohistochemistry results. The differences in clinicopathological factors between the two groups were assessed (Table 2). It was demonstrated that METTL14 expression was associated with pT stage (*p* < 0.01), pN stage (*p* < 0.01), M stage (*p* < 0.05), and TNM stage (*p* < 0.001). However, other factors such as sex, age, tumor location, tumor size, macroscopic type, and histologic grade were not related to METTL14 expression. Subsequently, we evaluated the METTL14 mRNA level in three GC cell lines (SGC‐7901, MGC‐803, and AGS) and GES‐1, and it was found that GC cell lines exhibited lower METTL14 mRNA levels than GES‐1 (Figure 1F). The findings above indicated that METTL14 was downregulated in both GC tissues and cell lines, and METTL14 might be associated with GC progression.

**Table 2:** Relationship between METTL14 expression and the clinicopathologic features of GC patients

Factors	Cases	METTL14 expression		*p*
		Low	High	
Sex				
Male	183	35	148	0.347
Female	65	16	49	
Age (years)				
≤60	134	22	112	0.080
>60	114	29	85	
Tumor location				
Upper	34	10	24	0.505
Middle	27	6	21	
Lower	130	23	107	
Entire	57	12	45	
Tumor size (cm)				
≤5	182	35	147	0.388
>5	66	16	50	
Macroscopic type				
Early stage/Borrmann I‐II	171	32	139	0.282
Borrmann III‐IV	77	19	58	
Histologic grade				
Differentiated	71	13	58	0.578
Undifferentiated	177	38	139	
pT stage				
T1–T2	74	6	68	0.002
T3–T4	174	45	129	
pN stage				
N0	91	10	81	0.005
N1–3	157	41	116	
M stage				
M0	239	46	193	0.020
M1	9	5	4	
TNM stage				
I–II	117	9	108	<0.001
III–IV	131	42	89	

Abbreviations: GC, gastric cancer; TNM, tumor‐lymph node‐metastasis.

### 3.2 METTL14 level and prognosis of GC patients

The relationship between the METTL14 level and the prognosis of patients who underwent surgical treatment was assessed. Survival analysis exhibited that patients with high METTL14 expression level showed a more favorable OS than patients with low METTL14 expression level (*p* < 0.001, Figure 1G). Prognosis of the GC patients was obviously correlated with tumor size (*p* < 0.001), pT stage (*p* < 0.001), pN stage (*p* < 0.001), M stage (*p* < 0.001), and METTL14 expression level (*p* < 0.001) in univariate analysis. Additionally, multivariate analysis exhibited that METTL14 high level was an advantageous prognostic factor, while pT stage (*p* < 0.001), pN stage (*p* < 0.001), and M stage (*p* < 0.001) were the independent adverse prognostic factors (Table 3).

**Table 3:** Univariate and multivariate analysis of prognostic factors in GC patients

Factors	Univariate analysis			Multivariate analysis		
	Cases	5‐YSR	*p*‐value	HR	95% CI	*p*‐value
Sex						
Male	183	71.7%	0.557	1.129	0.719–1.773	0.597
Female	65	57.7%				
Age (years)						
≤60	134	68.5%	0.617	0.820	0.207–1.112	0.831
>60	114	49.7%				
Tumor size						
≤5 cm	182	70.3%	<0.001	1.447	0.886–2.363	0.139
>5 cm	66	30.3%				
pT stage						
T1–T2	74	91.7%	<0.001	3.810	1.877–7.732	<0.001
T3–T4	174	46.3%				
pN stage						
N0	91	83.6%	<0.001	2.880	1.631–5.084	<0.001
N1–3	157	44.4%				
M stage						
M0	239	62.0%	<0.001	4.282	1.880–9.753	<0.001
M1	9	0.0%				
TNM stage						
I–II	117	63.9%	<0.001	2.775	1.466–5.255	<0.001
III–IV	131	25.7%				
METTL14 expression						
Low	51	25.5%	<0.001	0.463	0.305–0.704	<0.001
High	197	69.9%				

Abbreviations: 5‐YSR, 5‐year survival rate; CI, confidence interval; GC, gastric cancer; HR, hazard ratio.

### 3.3 METTL14 overexpression depresses GC cell growth and aggression in vitro

**FIGURE 2:**
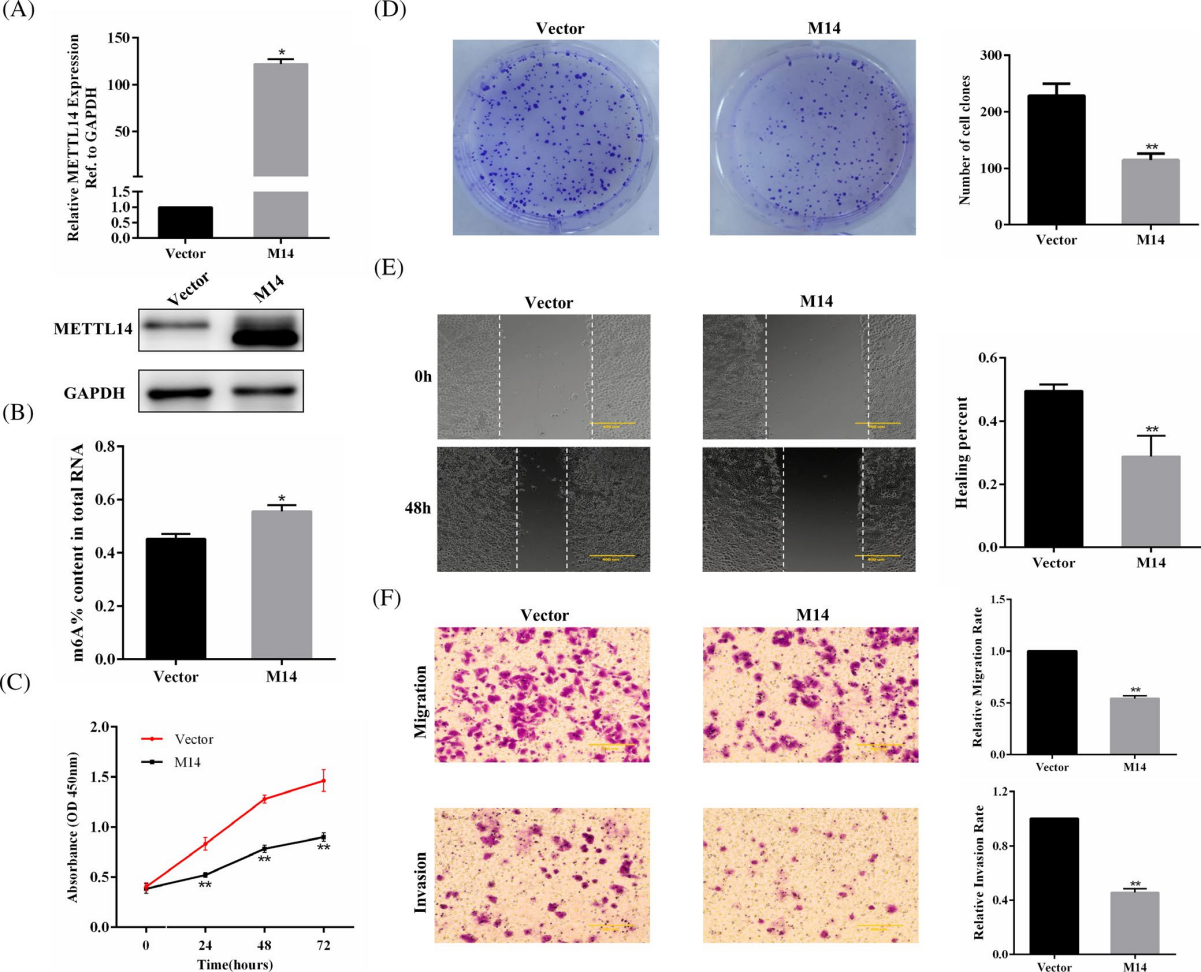
METTL14 overexpression inhibits SGC‐7901 cell proliferation and aggression in vitro. A, The efficiency of METTL14 overexpression in a stably transfected SGC‐7901 cell line measured by qRT‐PCR and Western blotting. B, The m6A contents of total RNAs in METTL14‐overexpressing SGC‐7901 cells. C, CCK‐8 assay for METTL14‐overexpressing SGC‐7901 cells. D, Colony formation assay for the treated GC cell line. E, Wound healing assay for METTL14‐transfected and Vector‐transfected GC cells. F, Transwell assays were employed to explore migration and invasion of the treated GC cell line. *p < 0.05, **p < 0.01

As the SGC‐7901 cell line exhibited the lowest METTL14 expression among the three GC cell lines, we transfected SGC‐7901 cells with lentivirus to construct a stable METTL14‐overexpressing cell line for further investigation. The transfection efficiency was evaluated (Figure 2A). The levels of m6A in the total RNAs of GC cells were then examined. The m6A level in SGC‐7901 cells was markedly increased after METTL14 overexpression (Figure 2B). To probe the effect of METTL14 on GC cell growth and aggression, a series of assays were conducted for each group. It was found that METTL14 overexpression notably retarded cell growth (Figure 2C). Besides, METTL14 overexpression significantly inhibited colony formation (Figure 2D). In the wound healing assay, upregulation of METTL14 suppressed the migration of GC cells (Figure 2E). Transwell assay indicated that METTL14 overexpression depressed GC cell aggression (Figure 2F). These findings suggested that METTL14 suppressed GC cell growth and aggression.

### 3.4 METTL14 depletion enhances GC cell growth and aggression in vitro

**FIGURE 3:**
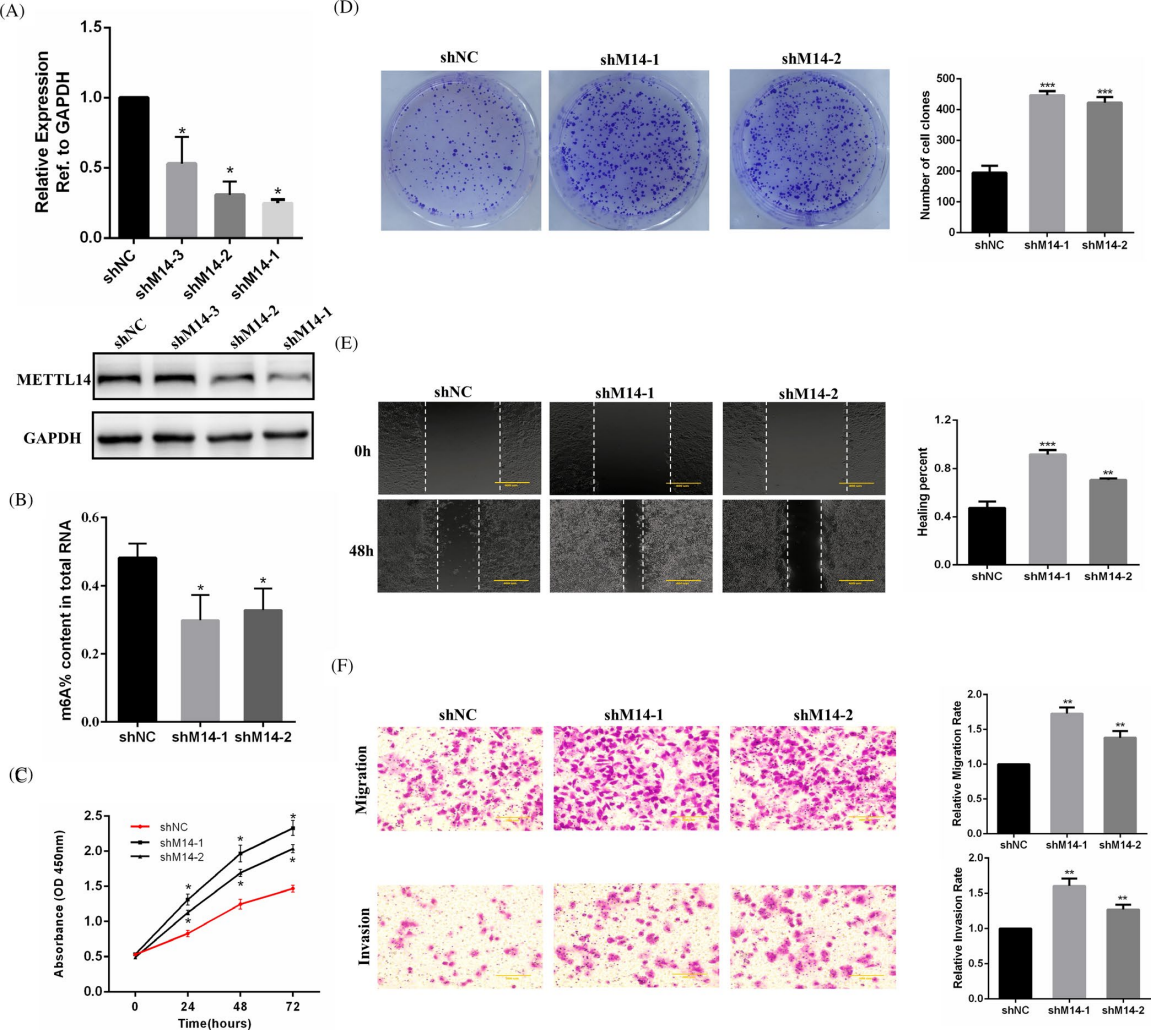
METTL14 depletion enhances AGS cell growth and aggression in vitro. A, The efficiency of METTL14 interference in a stably transfected AGS cell line evaluated by qRT‐PCR and Western blotting. B, The m6A contents of total RNAs in METTL14‐depletion AGS cells. C, CCK‐8 assay for the treated GC cell line. D, Colony formation assay in METTL14‐depletion AGS cells. E, Wound healing assay for METTL14‐depletion AGS cells. F, Transwell assays of METTL14‐depletion AGS cells. *p < 0.05, **p < 0.01, ***p < 0.001

The cell line AGS, exhibiting a high level of METTL14, was chosen to establish a stable METTL14‐silenced cell line. We obtained two cell lines with high transfection efficiency, namely, shM14‐1 and shM14‐2, which were used to carry out the subsequent experiments (Figure 3A). METTL14 depletion resulted in decreased m6A levels in GC cells (Figure 3B). The results of the cell proliferation assays demonstrated that METTL14 downregulation significantly promoted GC cell growth and colony formation (Figure 3C,D). In the cell aggression assays, METTL14 depletion promoted GC cell migration and aggression (Figure 3E,F).

### 3.5 METTL14 may participate in mediating the activation of the PI3K/AKT/mTOR pathway and the EMT pathway

**FIGURE 4:**
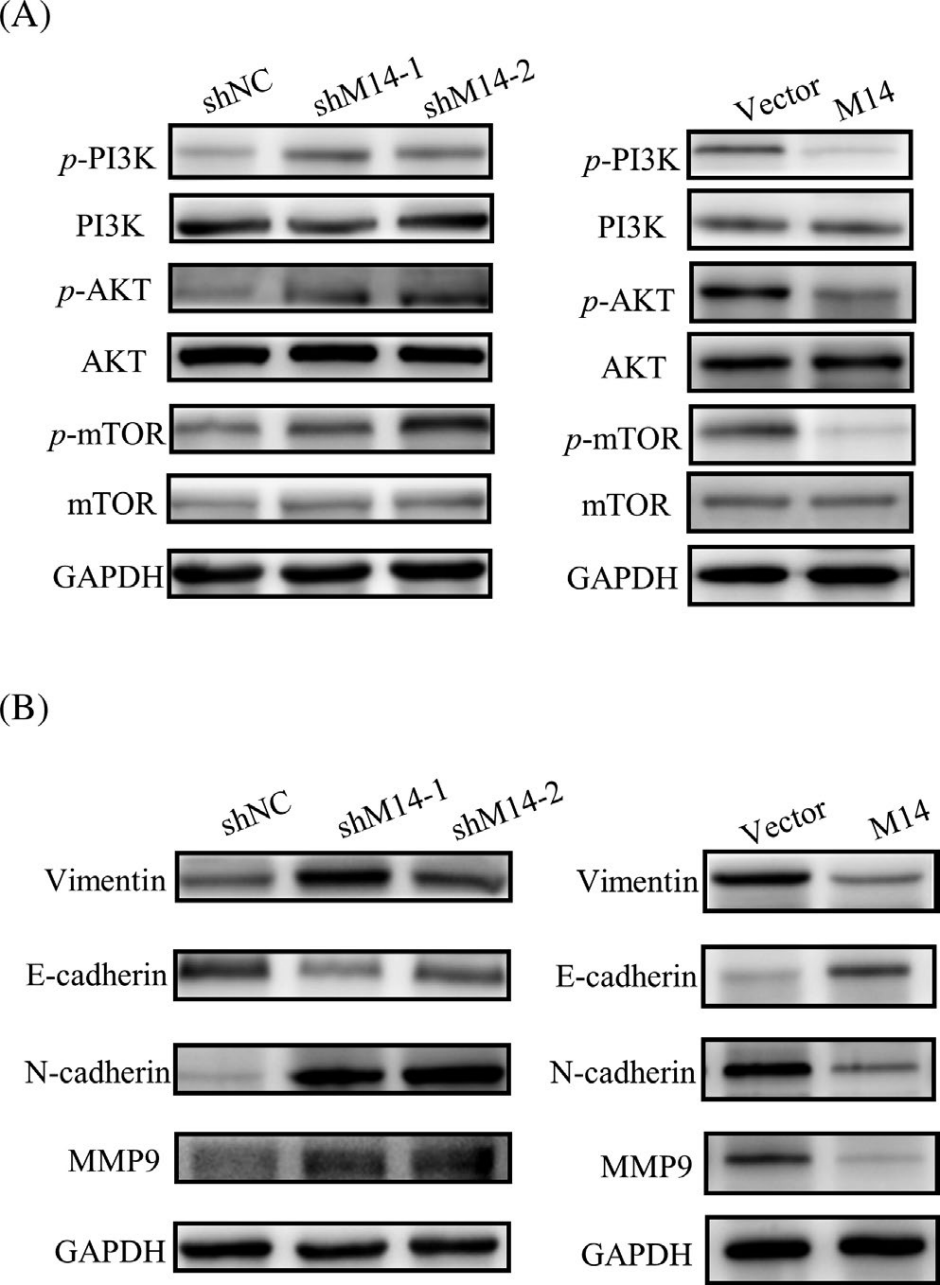
METTL14 may participate in mediating the PI3K/AKT/mTOR pathway and the EMT pathway. A, METTL14 affects the expression of the proteins related to the PI3K/AKT/mTOR pathway. B, Effects of METTL14 on the expression of proteins related to the EMT pathway

As reported, the PI3K/AKT/mTOR pathway was important for cell proliferation and development under physiological and pathological conditions. We then investigated the effects of METTL14 on the PI3K/AKT/mTOR pathway. It was demonstrated that METTL14 depletion increased phosphorylation of PI3K, AKT, and mTOR protein levels, whereas METTL14 overexpression downregulated the expression of the above proteins (Figure 4A). There was only a slight change in the expression of PI3K, AKT, and mTOR in each group. Our study confirmed that METTL14 could affect GC cell functions in migration and invasion; therefore, we probed the levels of proteins related to the EMT pathway. METTL14 downregulation elevated Vimentin, N‐cadherin, and MMP9 protein expression and decreased E‐cadherin protein expression, while METTL14 upregulation reduced Vimentin, N cadherin, and MMP9 protein expression and enhanced E‐cadherin protein expression (Figure 4B). Overall, the results above indicated that METTL14 overexpression could depress GC cell progression and aggression by deactivating the PI3K/AKT/mTOR pathway and the EMT pathway.

## 4 DISCUSSION

It has been reported that the downregulation of METTL3 induces apoptosis of HCC cells.^18^ Researchers have proven that FTO promotes cell proliferation of acute myeloid leukemia.^19^ The ALKBH5 protein maintains the stem cell characteristics and inhibits their differentiation ability in breast cancer.^20^ These novel findings suggest that RNA m6A modification and the related factors may regulate oncogenesis and progression of tumors. Therefore, exploring the biological functional role and potential molecular mechanism of m6A and the related factors has practical application value and medical prospects.

Here, we first observed that m6A modification levels were decreased in GC tissues compared with that in matched adjacent tissues. Using the Oncomine database, we speculated that METTL14 might be a key regulatory factor that caused m6A modification disorder in GC. We then confirmed that GC tissues expressed lower levels of METTL14 than paratumor tissues both in paired clinical samples and bioinformatics. The correlation between METTL14 expression and clinicopathological factors was further evaluated, and it was found that METTL14 expression was markedly correlated with TNM stage. Survival analysis proved that GC patients with high METTL14 expression had a better prognosis than those with low METTL14 expression. Multivariate analysis revealed that for GC patients, a high METTL14 level could be an advantageous prognostic factor. Combined with the results, we speculated that METTL14 might participate in the pathological processes related to GC cell proliferation and aggression. In the study, the METTL14 mRNA expression level differences between normal gastric tissues and gastric cancer tissues seemed negligible, while IHC study demonstrated significant difference in METTL14 protein expression between tumor and paratumor samples. The central dogma of biology tightly links DNA, RNA, and protein. After transcription, mRNAs undergo a series of intertwining processes to be finally translated into functional proteins. However, the mRNA abundance of a specific gene may not necessarily have a linear relationship with the protein expression of its translation product.^21^ There are many levels of gene expression regulation, and transcription regulation is one of the levels.^22^ Post‐transcriptional regulation, translation, and post‐translational regulation all play a role in the protein expression.^23^, ^24^, ^25^, ^26^ Moreover, mRNA abundance may be inconsistent with protein expression levels due to mRNA degradation, protein degradation, folding modification, and other factors.^26^, ^27^, ^28^, ^29^ Thus, it may explain the discrepancies between METTL14 mRNA expression and METTL14 protein expression in our study. However, the discrepancies between METTL14 mRNA expression and protein expression in the study are still not fully understood, and further studies are needed to further elucidate the issue. Subsequently, we examined the molecular effect of METTL14 on GC and found that METTL14 was the main molecule for the abnormal m6A modification in GC. Cell proliferation assays showed that GC cells with METTL14 depletion exhibited significantly enhanced abilities of proliferation and colony formation, while in GC cells with stable overexpression of METTL14, these two abilities were significantly weakened. Downregulation of METTL14 enhanced the aggression of GC cells, while upregulation of METTL14 depressed GC cell aggression. In summary, the results demonstrated that METTL14 might be considered as a tumor suppressor in GC.

METTL14, METTL3, and WTAP are three important components that constitute the methyltransferase complex.^30^ It was recently reported that METTL3 affected the proliferation of GC cells via deactivating the AKT signaling pathway.^31^ WTAP promoted the proliferation and migration of human ovarian cancer cells by affecting the activation of the AKT signaling pathway.^32^ Thus, we speculated that the three components of the functional methyltransferase complex might prefer the same target, AKT, to regulate downstream pathways. The PI3K/AKT/mTOR pathway is a pivotal regulatory pathway for cell proliferation and metabolism and is closely related to tumorigenesis and development.^33^ When receptor tyrosine kinases (RTKs) and G protein‐coupled receptors (GPCRs) are activated, PI3K phosphorylates phosphatidylinositol 3,4‐diphosphate to produce 3,4,5‐triphosphate phosphatidylinositol (PIP3). Activated PIP3 recruits and activates AKT in the cytoplasm, which in turn affects the level of related transcription factors involved in EMT and activates matrix metalloproteinase to promote cell invasion and metastasis.^34^ In our study, we evaluated the expression of proteins related to the PI3K/AKT/mTOR pathway to detect the potential mechanism by which METTL14 regulated the biological behavior of GC cells. Our results indicated that METTL14 exerted its function in GC cell lines by affecting the PI3K/AKT/mTOR pathway. However, it seems contradictory for METTL3, METTL14, and WTAP, the three components of the methyltransferase complex, to exhibit different effects on tumor cells. We speculated that the methyltransferases that determined cell fate might have tissue and cellular specificity.

Nearly half of GC patients who undergo surgical treatment develop tumor recurrence and metastasis, which are the main causes of death.^35^ EMT often occurs in the early stage of invasion and metastasis of tumor cells. During EMT, cells lose epithelial characteristics and obtain mesenchymal ones. The absence of the epithelial marker E‐cadherin and elevation of the mesenchymal marker Vimentin indicate the occurrence of EMT.^36^ EMT increases the migration and invasion of tumor cells, allowing tumor cells to infiltrate to surrounding stroma and resulting in the formation of a microenvironment that promotes tumor cell growth and metastasis.^37^ In our study, Vimentin, N‐cadherin, and MMP9 proteins were expressed at low levels in the METTL14 overexpression group, while E‐cadherin expression was high in this group. In the METTL14 depletion group, the expression of these proteins was reversed. These findings reveal a potential molecular mechanism whereby METTL14 interferes with the migration and invasion of GC cells via the EMT process.

In summary, our study indicates that METTL14 is the main regulator for the abnormal m6A modification in GC and METTL14 suppresses GC cell progression and aggression by deactivating the PI3K/AKT/mTOR pathway and the EMT pathway as a tumor suppressor. These findings elucidate that METTL14 may play a vital role in GC as a potential biological target.

## CONFLICT OF INTEREST

All authors declare that they have no conflict of interests.

## Supporting information

Tab S1

Tab S2

## Data Availability

All data used to support the findings of this study are available from the corresponding author upon request.
