# Supplementary material for: The m6A methyltransferase METTL14 inhibits the proliferation, migration, and invasion of gastric cancer by regulating the PI3K/AKT/mTOR signaling pathway
Source: J Clin Lab Anal. 2020 Dec 12;35(3):e23655. doi: 10.1002/jcla.23655 (PMC7957981; doi:10.1002/jcla.23655)
Supplement: Supplementary file 1 — Tab S1 [file JCLA-35-e23655-s001.docx]

**Supplementary Table S1** Information of two studies retrieved from the Oncomine database

| **Studies** | **Sample**  **size** | **Sample size of normal** | **Sample size of GC** | **Description** |
| --- | --- | --- | --- | --- |
| **TCGA gastric**  (http://gdac. broadinstitute. org/runs/ stddata__2013_05_23/ data/) | 637 | 330  (including 236 normal blood samples) | 307 | This dataset consists of Level 3 data (segmented using CBS) from the Broad Institute's Genome Data Analysis Center (GDAC). The resulting segments were mapped to RefSeq gene coordinates as provided by UCSC (UCSC refGene, July 2009; hg18, NCBI 36.1, March 2006). The samples were originally run on the Affymetrix SNP 6.0 platform. |
| **Deng gastric**  (http://www. ncbi.nlm.nih.gov/ geo/query/acc.cgi? acc=GSE31168) | 291 | 98 | 193 | Data was processed using AROMA.affymetrix. The resulting segments were mapped to hg18 (NCBI 36.1) RefSeq coordinates (UCSC refGene) as provided by UCSC (UCSC refGene, July 2009; hg18, NCBI 36.1, March 2006). The samples were originally run on the Affymetrix SNP 6.0 platform. |

All datasets were log-transformed and standard deviations were normalized to one per array.
