# Supplementary material for: The m6A methyltransferase METTL14 inhibits the proliferation, migration, and invasion of gastric cancer by regulating the PI3K/AKT/mTOR signaling pathway
Source: J Clin Lab Anal. 2020 Dec 12;35(3):e23655. doi: 10.1002/jcla.23655 (PMC7957981; doi:10.1002/jcla.23655)
Supplement: Supplementary file 2 — Tab S2 [file JCLA-35-e23655-s002.docx]

**Supplementary Table S2** The TCGA sample accession numbers of the RNAseq data downloaded from UCSC Xena

| **Sample accession numbers** | | |
| --- | --- | --- |
| \| TCGA-HU-A4GP-01 \| \| --- \| \| TCGA-FP-7735-01 \| \| TCGA-FP-7829-01 \| \| TCGA-IN-8462-01 \| \| TCGA-HU-A4GY-01 \| \| TCGA-BR-6852-01 \| \| TCGA-IN-7806-01 \| \| TCGA-BR-7704-01 \| \| TCGA-HU-8238-01 \| \| TCGA-BR-6454-01 \| \| TCGA-HU-A4GC-01 \| \| TCGA-HU-A4G3-01 \| \| TCGA-IP-7968-01 \| \| TCGA-BR-6458-01 \| \| TCGA-HU-A4GH-01 \| \| TCGA-HU-A4HB-01 \| \| TCGA-CG-5721-01 \| \| TCGA-CG-5720-01 \| \| TCGA-CG-5722-01 \| \| TCGA-BR-7851-01 \| \| TCGA-BR-7717-01 \| \| TCGA-IN-8663-01 \| \| TCGA-BR-7715-01 \| \| TCGA-BR-6564-01 \| \| TCGA-BR-6453-01 \| \| TCGA-BR-6457-01 \| \| TCGA-IN-AB1X-01 \| \| TCGA-BR-6802-01 \| \| TCGA-HU-A4GN-01 \| \| TCGA-BR-7716-01 \| \| TCGA-IN-AB1V-01 \| \| TCGA-CG-5734-01 \| \| TCGA-FP-A4BE-01 \| \| TCGA-BR-8081-01 \| \| TCGA-BR-4257-01 \| \| TCGA-RD-A8N6-01 \| \| TCGA-D7-8575-01 \| \| TCGA-VQ-A94P-01 \| \| TCGA-CD-A4MG-01 \| \| TCGA-IN-A6RO-01 \| \| TCGA-IN-A6RI-01 \| \| TCGA-IN-A7NU-01 \| \| TCGA-MX-A5UG-01 \| \| TCGA-VQ-A8DZ-01 \| \| TCGA-BR-8679-01 \| \| TCGA-VQ-A8P8-01 \| \| TCGA-VQ-A8P3-01 \| \| TCGA-VQ-A8PU-01 \| \| TCGA-CG-4465-01 \| \| TCGA-BR-8077-01 \| \| TCGA-BR-7703-01 \| \| TCGA-HU-A4H6-01 \| \| TCGA-BR-6452-01 \| \| TCGA-VQ-A91Q-01 \| \| TCGA-CD-5801-01 \| \| TCGA-BR-A4J6-01 \| \| TCGA-IN-A6RR-01 \| \| TCGA-BR-8381-01 \| \| TCGA-HU-A4GD-01 \| \| TCGA-VQ-A8PB-01 \| \| TCGA-BR-7722-01 \| \| TCGA-D7-6820-01 \| \| TCGA-VQ-A8PD-01 \| \| TCGA-BR-6563-01 \| \| TCGA-HU-A4GT-01 \| \| TCGA-MX-A5UJ-01 \| \| TCGA-BR-8365-01 \| \| TCGA-BR-6707-01 \| \| TCGA-VQ-A8E3-01 \| \| TCGA-D7-A4YV-01 \| \| TCGA-BR-4201-01 \| \| TCGA-BR-8588-01 \| \| TCGA-BR-6803-01 \| \| TCGA-HF-7136-01 \| \| TCGA-D7-6815-01 \| \| TCGA-BR-A4IU-01 \| \| TCGA-VQ-A922-01 \| \| TCGA-CG-5726-01 \| \| TCGA-VQ-A8E2-01 \| \| TCGA-CG-5725-01 \| \| TCGA-D7-A747-01 \| \| TCGA-VQ-A91Z-01 \| \| TCGA-F1-6875-01 \| \| TCGA-RD-A8NB-01 \| \| TCGA-BR-A4QL-01 \| \| TCGA-CD-8528-01 \| \| TCGA-VQ-A928-01 \| \| TCGA-D7-8579-01 \| \| TCGA-BR-4255-01 \| \| TCGA-VQ-A8PQ-01 \| \| TCGA-FP-8211-01 \| \| TCGA-CG-4449-01 \| \| TCGA-F1-6177-01 \| \| TCGA-VQ-A91S-01 \| \| TCGA-VQ-A91D-01 \| \| TCGA-CD-A48A-01 \| \| TCGA-BR-4191-01 \| \| TCGA-CG-4466-01 \| \| TCGA-BR-8297-01 \| \| TCGA-D7-6822-01 \| \| TCGA-D7-A4YX-01 \| \| TCGA-CG-4440-01 \| \| TCGA-D7-6525-01 \| \| TCGA-CG-5724-01 \| \| TCGA-D7-A4YY-01 \| \| TCGA-CG-4475-01 \| \| TCGA-BR-4367-01 \| \| TCGA-BR-A453-01 \| \| TCGA-R5-A805-01 \| \| TCGA-R5-A7ZF-01 \| \| TCGA-BR-A4IV-01 \| \| TCGA-BR-8369-01 \| \| TCGA-MX-A663-01 \| \| TCGA-EQ-A4SO-01 \| \| TCGA-CD-5813-01 \| \| TCGA-BR-6705-01 \| \| TCGA-R5-A7ZR-01 \| \| TCGA-BR-4253-01 \| \| TCGA-SW-A7EA-01 \| \| TCGA-BR-8676-01 \| \| TCGA-D7-A6F0-01 \| \| TCGA-BR-8059-01 \| \| TCGA-BR-8286-01 \| \| TCGA-BR-6456-01 \| \| TCGA-VQ-A8E0-01 \| \| TCGA-BR-8686-01 \| \| TCGA-VQ-A8PF-01 \| \| TCGA-FP-A4BF-01 \| \| TCGA-CD-8536-01 \| \| TCGA-D7-A4YT-01 \| \| TCGA-D7-8576-01 \| \| TCGA-CG-4442-01 \| \| TCGA-VQ-A8DT-01 \| \| TCGA-HU-A4G6-01 \| \| TCGA-D7-6528-01 \| \| TCGA-BR-A4IY-01 \| \| TCGA-BR-8284-01 \| \| TCGA-D7-A6EY-01 \| \| TCGA-CD-8526-01 \| \| TCGA-BR-8289-01 \| \| TCGA-MX-A666-01 \| \| TCGA-BR-4366-01 \| \| TCGA-BR-8484-01 \| \| TCGA-D7-A6EX-01 \| \| TCGA-BR-A4QI-01 \| \| TCGA-BR-8384-01 \| \| TCGA-IN-A6RJ-01 \| \| TCGA-KB-A93J-01 \| \| TCGA-CG-4306-01 \| \| TCGA-VQ-A8PK-01 \| | \| TCGA-D7-8574-01 \| \| --- \| \| TCGA-VQ-A8PP-01 \| \| TCGA-RD-A8MV-01 \| \| TCGA-BR-4368-01 \| \| TCGA-BR-8589-01 \| \| TCGA-VQ-A8PX-01 \| \| TCGA-VQ-A8P2-01 \| \| TCGA-BR-6706-01 \| \| TCGA-BR-A4PF-01 \| \| TCGA-CD-A489-01 \| \| TCGA-RD-A7BW-01 \| \| TCGA-RD-A8N2-01 \| \| TCGA-BR-A44T-01 \| \| TCGA-R5-A7ZI-01 \| \| TCGA-VQ-A91U-01 \| \| TCGA-D7-6526-01 \| \| TCGA-BR-7958-01 \| \| TCGA-CG-4438-01 \| \| TCGA-FP-A9TM-01 \| \| TCGA-BR-8080-01 \| \| TCGA-D7-A6EV-01 \| \| TCGA-CD-5798-01 \| \| TCGA-BR-4369-01 \| \| TCGA-HU-A4HD-01 \| \| TCGA-CG-4304-01 \| \| TCGA-BR-8485-01 \| \| TCGA-IN-A7NT-01 \| \| TCGA-CD-8527-01 \| \| TCGA-CG-4437-01 \| \| TCGA-HU-8249-01 \| \| TCGA-RD-A8MW-01 \| \| TCGA-BR-8362-01 \| \| TCGA-D7-6524-01 \| \| TCGA-VQ-A8PM-01 \| \| TCGA-BR-8683-01 \| \| TCGA-F1-6874-01 \| \| TCGA-D7-6521-01 \| \| TCGA-CG-4462-01 \| \| TCGA-VQ-A8PE-01 \| \| TCGA-VQ-A8DU-01 \| \| TCGA-D7-A4Z0-01 \| \| TCGA-VQ-AA6K-01 \| \| TCGA-D7-A748-01 \| \| TCGA-CG-4469-01 \| \| TCGA-VQ-AA68-01 \| \| TCGA-VQ-A927-01 \| \| TCGA-B7-A5TN-01 \| \| TCGA-BR-A44U-01 \| \| TCGA-BR-A4CR-01 \| \| TCGA-RD-A7C1-01 \| \| TCGA-BR-8361-01 \| \| TCGA-BR-8364-01 \| \| TCGA-D7-8573-01 \| \| TCGA-CG-4443-01 \| \| TCGA-D7-6519-01 \| \| TCGA-CG-5717-01 \| \| TCGA-BR-6565-01 \| \| TCGA-HU-8602-01 \| \| TCGA-BR-4267-01 \| \| TCGA-BR-4371-01 \| \| TCGA-BR-7196-01 \| \| TCGA-BR-8058-01 \| \| TCGA-HU-A4H0-01 \| \| TCGA-CG-4305-01 \| \| TCGA-BR-8682-01 \| \| TCGA-BR-8382-01 \| \| TCGA-BR-8363-01 \| \| TCGA-BR-8367-01 \| \| TCGA-BR-7959-01 \| \| TCGA-CG-4301-01 \| \| TCGA-BR-8371-01 \| \| TCGA-B7-5818-01 \| \| TCGA-BR-8380-01 \| \| TCGA-CD-8529-01 \| \| TCGA-VQ-A923-01 \| \| TCGA-CG-4436-01 \| \| TCGA-BR-8291-01 \| \| TCGA-FP-8209-01 \| \| TCGA-B7-A5TK-01 \| \| TCGA-RD-A7BS-01 \| \| TCGA-D7-6518-01 \| \| TCGA-VQ-AA64-01 \| \| TCGA-BR-4187-01 \| \| TCGA-BR-A4J1-01 \| \| TCGA-VQ-A91N-01 \| \| TCGA-HU-A4GJ-01 \| \| TCGA-VQ-A925-01 \| \| TCGA-FP-8099-01 \| \| TCGA-VQ-AA6I-01 \| \| TCGA-D7-6520-01 \| \| TCGA-CD-5804-01 \| \| TCGA-HU-A4GF-01 \| \| TCGA-BR-4361-01 \| \| TCGA-VQ-AA6J-01 \| \| TCGA-IN-A6RL-01 \| \| TCGA-HF-7133-01 \| \| TCGA-VQ-AA6B-01 \| \| TCGA-BR-8296-01 \| \| TCGA-VQ-A91W-01 \| \| TCGA-ZA-A8F6-01 \| \| TCGA-HU-8244-01 \| \| TCGA-BR-8590-01 \| \| TCGA-BR-8483-01 \| \| TCGA-ZQ-A9CR-01 \| \| TCGA-B7-A5TJ-01 \| \| TCGA-CD-8525-01 \| \| TCGA-D7-6818-01 \| \| TCGA-HU-A4GX-01 \| \| TCGA-CD-8530-01 \| \| TCGA-CD-8531-01 \| \| TCGA-BR-8373-01 \| \| TCGA-SW-A7EB-01 \| \| TCGA-IN-A7NR-01 \| \| TCGA-BR-A4J8-01 \| \| TCGA-R5-A7ZE-01 \| \| TCGA-HU-A4H8-01 \| \| TCGA-3M-AB46-01 \| \| TCGA-CD-A4MJ-01 \| \| TCGA-VQ-AA6F-01 \| \| TCGA-VQ-AA69-01 \| \| TCGA-BR-A4J4-01 \| \| TCGA-BR-A4J9-01 \| \| TCGA-FP-8210-01 \| \| TCGA-VQ-AA6A-01 \| \| TCGA-EQ-8122-01 \| \| TCGA-F1-A72C-01 \| \| TCGA-D7-6817-01 \| \| TCGA-CG-4476-01 \| \| TCGA-CG-5732-01 \| \| TCGA-BR-A4QM-01 \| \| TCGA-BR-8591-01 \| \| TCGA-BR-8486-01 \| \| TCGA-BR-4280-01 \| \| TCGA-RD-A7BT-01 \| \| TCGA-CG-4460-01 \| \| TCGA-HF-7131-01 \| \| TCGA-BR-6801-01 \| \| TCGA-VQ-A8PC-01 \| \| TCGA-HU-8604-01 \| \| TCGA-D7-8572-01 \| \| TCGA-CD-5800-01 \| \| TCGA-VQ-A94O-01 \| \| TCGA-VQ-A94T-01 \| \| TCGA-RD-A8N4-01 \| \| TCGA-HU-8608-01 \| \| TCGA-VQ-A91X-01 \| \| TCGA-KB-A93G-01 \| \| TCGA-D7-8570-01 \| \| TCGA-CD-8524-01 \| \| TCGA-R5-A7O7-01 \| | \| TCGA-CD-A4MI-01 \| \| --- \| \| TCGA-BR-4370-01 \| \| TCGA-VQ-A94R-01 \| \| TCGA-CD-8535-01 \| \| TCGA-BR-7707-01 \| \| TCGA-HU-A4GU-01 \| \| TCGA-CD-5799-01 \| \| TCGA-KB-A93H-01 \| \| TCGA-CG-4444-01 \| \| TCGA-BR-A4J7-01 \| \| TCGA-BR-4357-01 \| \| TCGA-BR-A4PE-01 \| \| TCGA-BR-8372-01 \| \| TCGA-RD-A8N9-01 \| \| TCGA-D7-A6F2-01 \| \| TCGA-VQ-A92D-01 \| \| TCGA-HU-8610-01 \| \| TCGA-CD-8533-01 \| \| TCGA-CG-4474-01 \| \| TCGA-CD-A487-01 \| \| TCGA-BR-A4J5-01 \| \| TCGA-BR-8487-01 \| \| TCGA-CG-5719-01 \| \| TCGA-BR-8687-01 \| \| TCGA-CG-5718-01 \| \| TCGA-F1-A448-01 \| \| TCGA-RD-A8N5-01 \| \| TCGA-BR-6566-01 \| \| TCGA-FP-7916-01 \| \| TCGA-BR-8368-01 \| \| TCGA-HU-A4G8-01 \| \| TCGA-BR-7957-01 \| \| TCGA-VQ-A8PH-01 \| \| TCGA-HU-8243-01 \| \| TCGA-VQ-A91E-01 \| \| TCGA-HF-7134-01 \| \| TCGA-VQ-AA6D-01 \| \| TCGA-D7-5577-01 \| \| TCGA-BR-A4CS-01 \| \| TCGA-FP-8631-01 \| \| TCGA-BR-7723-01 \| \| TCGA-3M-AB47-01 \| \| TCGA-B7-5816-01 \| \| TCGA-HU-A4G2-01 \| \| TCGA-IN-A6RS-01 \| \| TCGA-HU-A4G9-01 \| \| TCGA-CD-8532-01 \| \| TCGA-IN-7808-01 \| \| TCGA-BR-6455-01 \| \| TCGA-IN-A6RN-01 \| \| TCGA-BR-8366-01 \| \| TCGA-HU-A4H2-01 \| \| TCGA-CG-4477-01 \| \| TCGA-BR-A4IZ-01 \| \| TCGA-BR-4279-01 \| \| TCGA-VQ-A8E7-01 \| \| TCGA-D7-8578-01 \| \| TCGA-VQ-A8PJ-01 \| \| TCGA-CD-8534-01 \| \| TCGA-BR-8060-01 \| \| TCGA-VQ-A94U-01 \| \| TCGA-BR-7901-01 \| \| TCGA-FP-7998-01 \| \| TCGA-VQ-A91K-01 \| \| TCGA-VQ-A91V-01 \| \| TCGA-CG-5723-01 \| \| TCGA-BR-4362-01 \| \| TCGA-BR-8678-01 \| \| TCGA-HU-A4H3-01 \| \| TCGA-CD-A48C-01 \| \| TCGA-CG-4441-01 \| \| TCGA-BR-A4J2-01 \| \| TCGA-VQ-A91Y-01 \| \| TCGA-VQ-A8P5-01 \| \| TCGA-BR-4294-01 \| \| TCGA-BR-7197-01 \| \| TCGA-CD-5803-01 \| \| TCGA-D7-A4YU-01 \| \| TCGA-D7-5578-01 \| \| TCGA-CG-4472-01 \| \| TCGA-BR-4292-01 \| \| TCGA-FP-A8CX-01 \| \| TCGA-B7-A5TI-01 \| \| TCGA-CG-5716-01 \| \| TCGA-VQ-AA6G-01 \| \| TCGA-CD-A4MH-01 \| \| TCGA-D7-A74A-01 \| \| TCGA-CD-A486-01 \| \| TCGA-BR-4256-01 \| \| TCGA-BR-8592-01 \| \| TCGA-BR-4363-01 \| \| TCGA-RD-A8N0-01 \| \| TCGA-VQ-A8PO-01 \| \| TCGA-BR-8078-01 \| \| TCGA-BR-6709-01 \| \| TCGA-HF-7132-01 \| \| TCGA-D7-A6EZ-01 \| \| TCGA-HF-A5NB-01 \| \| TCGA-HJ-7597-01 \| \| TCGA-RD-A8N1-01 \| \| TCGA-BR-A452-01 \| \| TCGA-BR-8680-01 \| \| TCGA-VQ-A91A-01 \| \| TCGA-HU-A4H4-01 \| \| TCGA-VQ-A924-01 \| \| TCGA-HU-A4H5-01 \| \| TCGA-HU-A4GQ-01 \| \| TCGA-BR-8677-01 \| \| TCGA-D7-6522-01 \| \| TCGA-KB-A6F7-01 \| \| TCGA-BR-8690-01 \| \| TCGA-VQ-A8DV-01 \| \| TCGA-D7-6527-01 \| \| TCGA-BR-A4PD-01 \| \| TCGA-BR-8295-01 \| \| TCGA-CG-5733-11 \| \| TCGA-CG-5728-11 \| \| TCGA-CG-5730-11 \| \| TCGA-HU-A4GP-11 \| \| TCGA-FP-7735-11 \| \| TCGA-FP-7829-11 \| \| TCGA-IN-8462-11 \| \| TCGA-HU-A4GY-11 \| \| TCGA-BR-6852-11 \| \| TCGA-IN-7806-11 \| \| TCGA-BR-7704-11 \| \| TCGA-HU-8238-11 \| \| TCGA-BR-6454-11 \| \| TCGA-HU-A4GC-11 \| \| TCGA-HU-A4G3-11 \| \| TCGA-IP-7968-11 \| \| TCGA-BR-6458-11 \| \| TCGA-HU-A4GH-11 \| \| TCGA-HU-A4HB-11 \| \| TCGA-CG-5721-11 \| \| TCGA-CG-5720-11 \| \| TCGA-CG-5722-11 \| \| TCGA-BR-7851-11 \| \| TCGA-BR-7717-11 \| \| TCGA-IN-8663-11 \| \| TCGA-BR-7715-11 \| \| TCGA-BR-6564-11 \| \| TCGA-BR-6453-11 \| \| TCGA-BR-6457-11 \| \| TCGA-IN-AB1X-11 \| \| TCGA-BR-6802-11 \| \| TCGA-HU-A4GN-11 \| \| TCGA-BR-7716-11 \| \| TCGA-IN-AB1V-11 \| \| TCGA-CG-5734-11 \| |
